# Marginal quality of a full-body bulk-fill composite placed with an universal adhesive system in etch-and-rinse and self-etch mode: An *in vitro*study

**DOI:** 10.4317/jced.58081

**Published:** 2021-08-01

**Authors:** Antonio Signore, Luca Solimei, Marianna-Georgievna Arakelyan, Alina-Vladimirova Arzukanyan, Nicola De Angelis, Andrea Amaroli

**Affiliations:** 1 Department of Therapeutic Dentistry, Institute of Dentistry, I.M. Sechenov First Moscow State Medical University, Moscow, Russian Federation; 2 Department of Surgical and Diagnostic Sciences (D.I.S.C.), University of Genoa, Genoa, Italy; 3 Department of Orthopaedic Dentistry.Institute of Dentistry, I.M. Sechenov First. Moscow State Medical University. Trubetskaya str., 8, b. 2, 119992. Moscow, Russian Federation

## Abstract

**Background:**

Marginal seal of a nanohybrid bulk-fill composite compared to a nanohybrid conventional composite, using a universal adhesive (UA) applied in etch-and-rinse (ER) and self-etch (SE) mode was investigated.

**Material and Methods:**

Thirty-six intact molars were selected and two standardized cavities in each tooth were prepared and allocated into four groups according to restorative material and etching strategy. All samples were placed in a 1% methylene blue solution for 24 h, then cut in the middle of the restorations obtaining two parts (n=144) and used for microscopic evaluation (50x) for dye penetration measurements.

**Results:**

The data were analysed by ANOVA and Tukey post-hoc test (*p*<0.05). Marginal seal was influenced by adhesive strategy (*p*<0.05) but not from the composite used (*p*>0.05).

**Conclusions:**

Simplified restorations with nanohybrid bulk-fill composite showed comparable marginal leakage to incrementally placed nanohybrid composite. The UA used with a ER technique resulted in better marginal seal irrespective of the restorative material used.

** Key words:**Bulk-fill composite, universal adhesive, self-etching, etch-and-rinse, marginal seal.

## Introduction

Among the essential factors determining clinical success of resin based direct restorative materials are the marginal seal and absence of leakage (1,2). However, reliable adhesion can be compromised by the polymerization contraction stress occurring when the composite resin is placed in a prepared cavity and adhesively bonded to preparation walls. To control the polymerization stress of dental composites is thereby a kay factor to ensure proper marginal integrity and consequently longevity of the restoration (3). The factors that influence polymerization shrinkage have been widely investigated, nevertheless is highly complex and difficult to quantify (4). Previous studies have revealed that the entity of the polymerization stress depends on a combination of material characteristics and cavity design, as well as the restoration’s volume, compliance of the bonding substrate and the utilized restorative techniques (5,6).

A relevant role for interfacial stress development has been ascribed to cavity geometry. A high C-factor, namely the cavity conFiguration, due to a larger number of bonded surfaces, is an significant element in the onset of polymerization stress (7).

Therefore, several strategies have been recommended to reduce shrinkage stress, particularly in extensive composite restorations. Resins with a lower modulus of elasticity as an intermediate elastic layer of 0,5-1mm between the dentin substrate and the restoration materials has been suggested (8) A thicker adhesive bonding layers or a flowable composite as base material were suggested for absorbing a certain amount of stress (9). In addition, modified light-curing protocols, such as soft-start methods or pulsed light curing to reduce stress have been introduced during the last decade (10,11).

Not the least, aiming to reduce the C-factor, ensuring adequate diffusion of the curing light and to optimize composite internal marginal adaptation, several incremental layering techniques were recommended (12,13). Aim of these clinical procedures are reduce the final volumetric shrinkage of the composite resin, thus the level of polymerization shrinkage stress generated, and therefore to minimize marginal and internal gap formation (14).

In recent years extensive efforts have also been made on developing resin composite with low stress behavior trough changing monomer’s structure or chemistry, increasing the molecular weight, enhancing the filler amount with nanometric fillers or modifying initiation systems (13,14). This efforts has led to the introduction of innovative so called “bulk-fill” composite, which can be applied in bulk up to a thickness of 4 or 5 mm in direct posterior restorations do not require any incremental technique (14,15). Their resulting volume analysis and shrinkage stress showed a significant reduction in polymerization shrinkage stress and an adequate curing at a 4-mm thickness has been reported (16,17). Actually two different types of bulk-fill composite resins are commercially available: the full-body bulk-fill and the base, also referred to as flowable bulk-fill resin composites. The full-body bulk-fill have a high inorganic filler loading, resulting in a high viscous consistency. For this reason they are also referred to as paste-like or sculpTable bulk-fill resin composites. Their resulting volume analysis and shrinkage stress showed promising results compared with regular methacrylate composites, the flexural strength and wear resistance has been compared to that of conventional composite resins (15,16). This may support the intended use of these materials for bulk filling, in areas of high functional load in deep cavities and for high C-factor application (13).

As several *in vitro* and *in vivo* research have investigated the properties and clinical outcome of various new composites resins for bulk application, among the many factors involved in gap formation, the performance of adhesive systems constitutes a significant factor affecting the marginal quality (18,19).

Contemporary dental adhesive systems can be classified into two main categories according to different bonding strategies to dental substrates, the etch and rinse (ER) and the self-etch (SE) adhesive systems (1).

Recently some manufacturers introduced more versatile adhesive systems, that enables the practitioner to choose which adhesive strategy to use, indistinctly ER mode, SE mode or an alternative enamel selective etching (ESE) strategy, which actually is a combination of ER approach on enamel and SE approach on dentin (20,21). Current UAs are simplified adhesives, such as two-step ER or, most of them, one-step SE adhesives, represents the newest typology of adhesives on the market and, according to the respective manufacturers, allows the clinician to decide on a dedicated adhesive protocol depending on the clinical situation (21,22). They basically differ in their aggressiveness according to the pH of the acidic monomers, similarly to SE adhesives, thus ultra-mild (pH≥2.5), mild (pH≈2) and intermediately strong (pH between 1 and 2) (23,24). Acidic functional monomers are the principal ingredient of recently developed UAs, as they play a major role in Chemical adhesion to tooth structures of recently developed UAs is promoted by acidic functional monomers (25).

Based on the findings in the literature, UAs have shown interesting results in both, laboratory evaluations and clinical trials (26-28). Earlier studies were conducted to evaluate the adhesive performance of UAs using different types of direct restorative materials, including microhybrid, nanohybrid composite and nanocomposite, but despite promising results, there is a lack of data using UAs with bulk-fill composites (22,26,29). In previous studies mostly either ER or SE bonding systems have been used in order to reduce the variability in the results, however the need of further investigation of the relationship between the bonding agent and the bulk-fill composites has been recently advocated (30,31).

To the extent of our knowledge, there is no reporting research in the literature assessing the degree of marginal microleakage of bulk-fill composite resins used in combination with UAs. Therefore, the purpose of this study was to compare under *in vitro* condition the early sealing ability of a nanohybrid bulk-fill composite compared to a nanohybrid non-bulk composite, using a UA applied in SE and ER mode. The null hypothesis tested were that: 1) there is no statistically significant difference between the two application mode of the same UA neither in SE nor in ER mode 2) neither the nanohybrid bulk-fill composite nor the conventional nanohybrid composite under investigation show a statistically significant difference in microleakage, when same adhesive strategy was applied.

## Material and Methods

-Specimen preparation

In total thirty-six intact, non-carious, unrestored, permanent maxillary and mandibular molars, recently extracted for periodontal reasons, were selected for this *in vitro* study. The teeth were collected after obtaining the respective patient’s informed consent. The privacy rights of the patients have been always observed. Right after extraction, the remaining connective tissue and debris were removed by means of ultrasonic devices and rinsed with distilled water. Then the teeth were stored in 0.5% chloramine T aqueous solution at 4°C and used within 3 mos of extraction.

All procedures were performed by two experienced operators with the use of 4.3x400 surgical head-worn loupes (KS, Carl Zeiss Vision, Germany). Using a calibrated cylindrical diamond bur (80 μm) the teeth were prepared at a speed of 40,000 rpm under air-water cooling. On both sides of the molars, buccal and lingual/palatal, a square cavity was prepared with the following dimensions: 4 mm depth 4mm (measured along the lateral wall), 4 mm width (pulpal wall) and 4 mm length (buccal or lingual wall). All margins were confined to the enamel and butt-joint finished with fine-grit diamond bur (25 μm). The cavity dimension of each sample was measured using a digital sliding calibre (PAV, Göttingen, Germany). In this way standardized, box-shaped preparations were obtained, having a cavity conFiguration factor (C-factor) of 5.0 and an approximate volume of 64 mm3. Later in the process, after sectioning, measurements of the cavity dimensions were performed by means of an image analysis software under a stereomicroscope (see the “Microleakage analysis” section). Totally seventy two cavities have been prepared for restorative procedures.

-Bonding Procedures

The teeth were randomly subdivided into four equal groups of nine teeth each according to the materials to be tested:

Group 1: Universal adhesive Futurabond U (FbU) (VOCO; Cuxhaven, Germany) in SE mode/Bulk-fill nanohybrid composite Admira Fusion x-tra (AFx-tra) (VOCO; Cuxhaven, Germany) 

Group 2: FbU in ER mode/AFx-tra

Group 3: FbU in SE mode/Nanohybrid composite Admira Fusion (AF) (VOCO; Cuxhaven, Germany) 

Group 4: FbU in ER mode/AF

The composition of the tested materials is mentioned in Table 1. For group 1 and 3 the universal adhesive FbU was used in the SE mode according to manufacturer’s instructions and an active application of the bonding was performed. The same bonding agent was used in an ER mode for both group 2 and 4 following manufacturer’s instructions: The enamel and dentin of each cavity have been etched with 35% orthophosphoric acid (Vococid, VOCO; Cuxhaven, Germany) for 30 s and 10 s, respectively. After rinsing for 15 s, the water excess was removed paying attention not to dehydrate dentin and an active brushing of the bonding was also applied. For each group the adhesive was light-cured according to manufacturer’s instructions for 10 s under a LED light curing unit (VALO Cordless, Ultradent Products, Inc., South Jordan, UT, USA) spectrum 395–480 nm, with an output energy of 1.000mW/cm2. The same LED curing unit with the same settings was used for light-curing the restorative materials. After the bonding procedures, the cavities of group 1 and 3 were filled in a single increment with the bulk-fill nanohybrid composite AFx-tra shade universal according to manufacturer’s instructions and polymerized for 20 s. The nanohybrid composite AF shade A2 was applied in the cavities of group 2 and 4 in two horizontal increments (2 mm) and separated light-cured for 20 s following manufacturer’s instructions. The light output of the curing unit was measured using a LED radiometer (Demetron; Kerr, Orange, CA, USA) after completing five specimens. Totally seventy two restorations have been performed, two on each tooth, thirty-six per each tested restorative material.

**Table 1:**
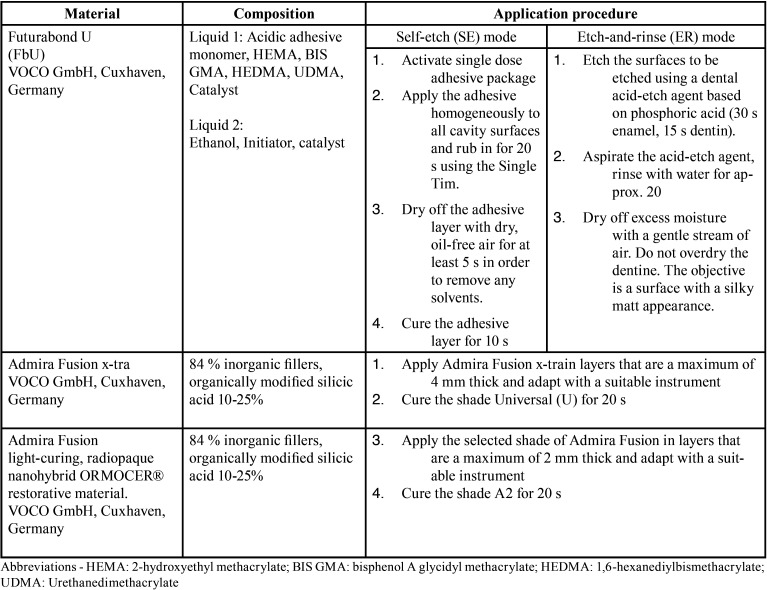
Materials, composition and application procedure according to the information supplied in the safety data sheets and manufacturer’s instruction.

The finishing procedure was done by means of graded abrasive discs (Sof-Lex, 3M ESPE, Seefeld, Germany). Finally, the polishing procedure was carried out using diamond and aluminum oxide three-step pastes (ENA Shiny, Micerum SPA, Avegno, Italy). The first 3-μm and 1-μm diamond pastes (Shiny A and B, Micerium SPA, Avegno, Italy) were applied sequentially with goat hair brushes, and then the aluminium oxide paste (Shiny C, Micerium Spa, Avegno, Italy) was applied with cotton felt. Between each step, the tooth surface was cleaned with a gauze soaked in alcohol to eliminate oils contained in the polishing pastes. Afterwards, the restored teeth have been kept in distilled water at room temperature (20 to 23°C) for 24 h.

-Microleakage analysis

Specimens were not subjected to cycling loading or thermocycling, so that the effect of the immediate bonding procedure alone could be assessed. The teeth were dried and the samples were completely coated with two layers of nail varnish based on acetone, leaving 1 mm frame around the four cavity margins. To ensure the varnish to dry, the specimens were left for 3 h at room temperature. The samples were then placed for 12 h in normal saline solution to rehydrate the teeth desiccated tissues. The prepared specimens of each group were placed in a 1% methylene blue solution for 24 h at room temperature, after which the tooth surfaces were cleaned from the dye by means of silicone finishing and polishing rubbers. Samples sections of the prepared samples were cut by means of a low speed precision sectioning saw (IsoMet, Buehler, Lake Bluff, Illinois, USA) in the middle of the height of restorations parallel to the occlusal surface, obtaining two sections (A and B), i.e. equal halves of the restorations, both considered for statistical analysis. Each section, a total of hundred forty-four, have been polished by means of a grinder polishing lapping machine (PLANOPOL-2, Struer, Copenhagen, Denmark) and used for microscopic evaluation.

All the sections were maintained moist until the microscopic observations took place, then dried with absorbent paper. For the evaluation of dye penetration pictures of the restoration interfaces were taken under 50x magnification by means of a stereo microscope (Wild Heerbrugg M5A, Wild Heerbrugg AG, Switzerland). In each sample the depth of dye penetration was evaluated along both side walls (mesial and distal) and assessed by means of an image analysis system (Leica Q500IW, Leica Microsystems, Wetzlar, Germany) to the total extension of the restoration interface. The marginal infiltration was measured for both restoration walls, mesial (M) and distal (D), of the two tooth sections. It follows that each tooth has allowed to perform eight marginal leakage analysis, four for each section, for a total amount of two hundred eighty-eight considered for statistical analysis.

The scoring system used to quantify interfacial microleakage was codified using a scale as follow:

Score A: no infiltration (0 mm)

Score B: infiltration ≤0,2 mm

Score C: infiltration 0,2÷0,5 mm

Score D: infiltration 0.5÷1 mm

Score E: infiltration ≥1 mm

The images were analyzed separately by two experienced evaluators. In case of disagreement, differences in scoring were discussed until a final value was assigned on consensus.

-Statistical analysis

To evaluate the effects of application mode of the universal adhesive (SE and ER) and nanohybrid composites (AF, AFx-tra) and their interaction on marginal infiltration within each group, one-way analysis of variance (ANOVA) was performed. Subsequently, a Tukey Honestly Significant Difference (HSD) post-hoc test was applied on means and standard deviations (SD), to indicate which groups were significantly different from others. Differences were considered statistically significant for *p*<0.05.

## Results

Measurements of the cavity dimensions revealed that the widths and depths of the interfaces were not statistically significant different among all groups, (*p* >0.05).

Table 2. summarizes the mean dye penetration scores through the tooth-composite interface expressed in mm (mean±SD) measured for each group of treatment, and the conversion to percentage of the total gap formation length as a function of total length of the interface.

**Table 2:**
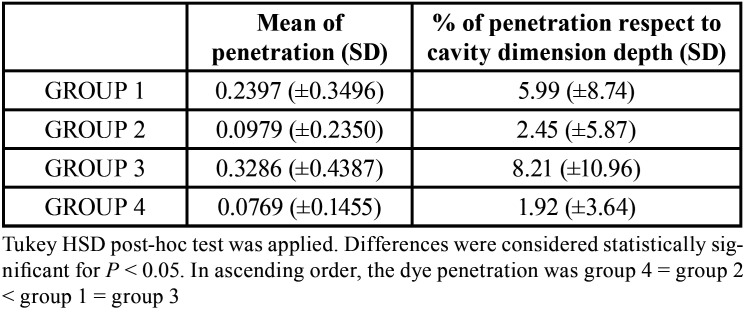
Show the average±standard deviation of the marginal infiltration by measures (mm) of each restoration wall, mesial (M) and distal (D), of both tooth sections, for each group of treatment (72 measures for each group). The table show also the % of penetration respect to cavity dimension depth of 4 mm. Group 1: Universal adhesive Futurabond U (FbU) in SE mode/Bulk-fill nanohybrid composite Admira Fusion x-tra (AFx-tra); Group 2: FbU in ER mode/AFx-tra; Group 3: FbU in SE mode/Nanohybrid composite Admira Fusion (AF); Group 4: FbU in ER mode/AF.

The group 1 shows a dye penetration higher than the group 2 [0.2397 (±0.3496) vs 0.0979 (±0.2350)] with a statistically significative difference (*p*<0.05). Conversely, the group 1 and the group 3 [0.2397 (±0.0.3496) vs 0.3286 (±0.4387)] as well as the group 2 and the group 4 [0.0979 (±0.4933) vs 0.0769 (±0.1455)] have no statistically significative difference (*p*>0.05). However, the treatment of the group 4 significantly allow lesser dye penetration respect to the group 1 and 3 (*p*<0.01 and *p*<0.001, respectively). Lastly, the dye penetration was statistically lower (*p*<0.001) in group 2 respect to the group 3. According to this results, the type of filling technique and filling material had no statistically significant influence on the results; at the same time statistically significant differences were observed between the application mode of the universal bonding system. Therefore, in ascending order, the difference in the marginal dye penetration was group 4 = group 2 < group 1 = group 3.

The number and percentage of marginal infiltrations for each experimental group and the dye penetration rating is reported in Table 3. The highest rating (score A, no dye penetration) was achieved by 70.9% of the restorations made of FbU in ER mode/AFxtra; 66.7% of restorations FbU in ER mode/AF; 54.2% of restorations of the composite FbU in SE mode/AFx-tra; and 41.6% of restorations of FbU in SE mode/AF. In restoration groups FbU in SE mode/AFx-tra and FbU in SE mode/AF only, the lowest rating (score E, >1 mm dye penetration) was recorded in 8.3% of the specimens.

**Table 3:**
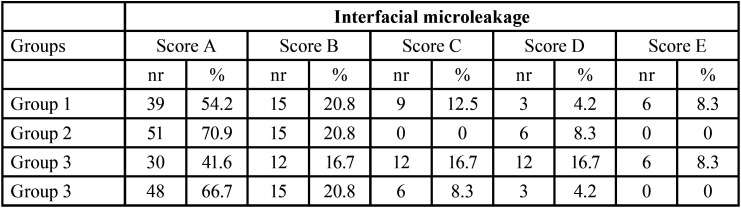
Summarizes the dye penetration rating and shows the number and percentage of infiltrations for each experimental group. Group 1: Universal adhesive Futurabond U (FbU) in SE mode/Bulk-fill nanohybrid composite Admira Fusion x-tra (AFx-tra); Group 2: FbU in ER mode/AFx-tra; Group 3: FbU in SE mode/Nanohybrid composite Admira Fusion (AF); Group 4: FbU in ER mode/AF. The scoring system used to quantify interfacial microleakage was codified using a scale as follow: score A: no infiltration (0mm); score B: infiltration ≤0.2mm; score C: infiltration 0.2±0.5 mm; score D: infiltration 0.5±1mm; score E: infiltration ≥1mm

## Discussion

Based on the results data of the present study, the first formulated null hypothesis had to be rejected as significant differences in microleakage emerged among the groups, given that the dye penetration scores of group 2 and 4, in which the universal bonding was used in ER mode differed significantly from those recorded in the other two groups, in which the universal bonding was applied in SE mode. The UA used in this study applied in ER mode resulted in significantly better marginal seal then the SE mode did, irrespective of the restorative material placed. Taking into consideration, that there is a lack of data assessing the degree of microleakage of bulk-fill composites used in combination with universal adhesives, such evidence can be compared with previous findings (21,32,33). Several *in vitro* studies, in which however conventional high-viscous resin composites have been used, reported significantly improved enamel bond characteristics and marginal integrity when ER strategy or ESE was utilized for UAs (34,35). This may be attributed to the enamel morphological patterns that have been formed after etching with phosphoric acid, that promotes a deeper enamel demineralization, therefore the increase of the surface area and wettability, thus increasing micro-mechanical interlocking (36). In this context, when UAs are used with the SE strategy, the lower acidity compared with phosphoric acid, reduces their effectiveness to demineralize the enamel surface, thereby resulting in mild micro-retentive porosities and subsequent provision of micromechanical retention (29). Following the acidity classification of SE adhesives, the acidity of Futurabond U, the universal bonding agent tested in the present study, is pH 2.3, therefore can be considerate mild (37,38). For UAs, the disadvantage of the SE protocol which was observed, implies that application UAs in SE mode on the enamel is associated with some concerns (22). As a result, ESE was considered the best strategy for optimizing adhesive performance to enamel and improving marginal seal (21).

In addition to that, *in vitro* studies have suggested several ways to improve the resin–enamel bond performance with UAs. One of this indicated that a double application of UAs may be efficient in enhancing enamel bond strengths with the SE mode, possibly due to the improved mechanical properties of the thicker adhesive layer (39). Further studies have suggested to use a prolonged and active application of the UA with the SE strategy as a viable alternative to enamel etching (40,41). Consideration should be also given to the possibility of reducing marginal leakage with the use of an additional resin layer after application of an UAs used in the ER or in the SE modes. It has been demonstrated, that the use of a hydrophobic resin coat may be beneficial, however the reduction in marginal leakage could be mainly related to the adhesive composition, than on the bonding strategy (42,43). However, this adhesive application strategies have been not applied in the present investigation, and no scientific information is so far available regarding this adhesive application modes using bulk-fill composites. Further studies are required to elucidate this topic better.

Even if to date, there are is a lack of data assessing the degree of microleakage of high-viscosity bulk-fill composite resins used in combination with UAs utilized in SE or ER mode, several other *in vitro* studies have reported good marginal quality when ER strategy was utilized in combination with bulk-fill composite materials (14,44,45). Based on our observations, all experimental groups presented the best score, namely no dye penetration, but only the two groups, 2 and 4, using the UA in ER mode reached a significant high percentage, 70.9 and 66.7 respectively.

For instance, very newly a meta-analysis, evaluating the marginal integrity of bulk fill composites, confirms that where both SE and ER technique was used, ER adhesives performed better than SE adhesives (46). Recently the interface integrity of a full-body bulk- fill composites to enamel and dentin has been tested with a 3-step ER and a 1-step SE adhesives, observing a better marginal sealing of the ER bonding system compared to the 1 SE bonding system irrespective of the bulk-fill composite tested (31).

In contrast to those findings, satisfactory interfacial seal in class II and class V restorations was reported by new bulk fill resin composite at the enamel interface with the use of a single-step SE adhesive (47,48). In this regard it interesting to note that in the present study satisfactory marginal seal, such as no infiltration and an infiltration ≤0,2 mm (pooling the score A and B) was obtained in a considerable percentage even for the two experimental groups, in which the UA tested was used in SE mode, such as 75% for group 1 and 58.3% for group 3.

At this point it could be conceivable that a weaker adhesive performance of UAs used with SE mode could be compensated by a reduction of polymerization stress of bulk-fill composites (31). A plausible assumption that bulk-fill materials with reduced polymerization shrinkage stress might positively affect marginal seal of a SE application mode of UAs ,could not be confirmed by our results. Such scenario is supported by other data previously collected and expands the findings that saw a better performance of ER strategy in marginal seal (14,19).

The second null hypothesis, which anticipated no significant differences in microleakage among the nanohybrid bulk-fill composite and the conventional nanohybrid composite under investigation, was accepted, because the type of filling material and filling technique had no statistically significant influence on the results. We verified that marginal quality of the bulk-fill composite placed in one increment was similar to that of the conventional composite resin placed in two increments, when same adhesive strategy was applied. Our results are consistent with the findings from other studies, which have reported, that the dye-penetration measurements of the investigated bulk-fill composites performed similarly to a well-established conventional incrementally placed composite in withstanding the shrinkage stresses (19,30,49). The single-increment, bulk-up application and polymerization method carried out in the present study did not affect negatively marginal adaptation of the restorations. For the two experimental groups, in which the bulk-fill composite tested was used (group 1 and 2) the dye penetration test showed no infiltration and an infiltration ≤0,2 mm (therefore considering score A and B together) in a high percentage of tested restorations 75.00% and 91.70% respectively. The evidence that emerges from our results, complements the data previously collected on the quality of marginal seal of a nanohybrid bulk-fill material, when the same adhesive strategy was applied (44,50).

Since our objective was to investigate the marginal sealing ability of the investigated materials, the cavity design and conFiguration had to be comparable between the experimental groups, in order to eliminate the cavity as a variable that may influence the results. A cavity dimension 4 mm depth, 4 mm width and 4 mm length, even if performed on buccal and lingual/palatal aspect of the samples, may reflect the dimensions of a primary medium-sized direct restorations performed by general practitioners. Likewise allowing standardization in cavity design and cavity configuration, compared to a typical class I or class II cavity. Among the latter, natural diversity in tooth morphology, sizes and shapes are responsible of ineviTable variations in the results, which can limit the power of a systematic review (49,51).

Experimental testing conditions hardly permit use of restorative cavities where C-factors were identical (52). We verified, though, that there were no statistically significant differences in wall dimensions of the restorations or between groups. It can be assumed, that the overall result of the present study was not influenced by the cavity design significantly. Moreover, all margins have been located on enamel only, to due to eliminate the marginal substrate as additional variable that may influence the results. However, most resent *in vitro* studies offers particular attention to the marginal quality of class II restorations performed with bulk fill resin composite in one increment compared to non-bulk composites placed in several increments (14,51). For class II and all the more reason MOD cavities, the outcome of the marginal seal, may be a consequent of the cuspal deflection (53,54). The vertical slot cavity used in this study, conversely is a more rigid model with a limitation of the cavity walls movements. In contrast to our results, a lower degree of dye penetration in fillings made with layering technique, than with the single-increment technique has been observed in previous works (55). On the other hand, the marginal quality of Class II restorations of composite resins placed in one increment was observed as similar to that of restorations placed in several increments (13,14). In this respect, our results further support the outcome of previous investigations, that the simplified restorations with bulk-fill composite showed similar marginal quality to incrementally placed traditional composites (19,47,56).

Another possible explanation for the absence of significant differences in microleakage among the nanohybrid bulk-fill composite and the conventional nanohybrid composite under investigation, may be related to the use of an UA of the same manufacturer. Considering the data from previous *in vitro* studies on composite resins placed in single or in several increments in combination with UA systems, it was advocated that the performance of multi-mode adhesives was dependent not only on the adhesive approach. Based on a systematic review, other factors, such as pH, application method and adhesive composition could influence the leakage of UAs (24). Commonly UAs have the same versatility of being used in both SE and ER strategies, however the differences in their monomer composition might be the reason for their different performance (57). Different reports have emphasized, that within the same adhesive system produced by the respective manufacturer, the type of filling material and filling technique did not significantly influenced the results, indeed the percentage of regular margin was not affected by either the incremental technique or the investigation method (19,52,54). Therefore, in the present study the choice of testing both composite materials in combination with an adhesive proposed by the same manufacturer was made to prevent possible incompatibility phenomena between adhesive and the composites that might affect the results. However, this relationship between a compatible bonding agent and the bulk-fill composite offered by manufacturer as integrated systems requires further studies.

The experimental set-up used in this study had several limitations that deserve some comments. In the present investigation only products from one manufacturer have been used. As already mentioned the results in marginal quality may have been different if bonding agent and composite resins from different manufactures had been used. It should also be pointed that our results were gained in the *in vitro* conditions such as an easy and direct access to the prepared tooth samples, as well as an optimal capability of light-curing device. The distance between the tip end of the curing unit and the irradiated restorations’ surface achieved experimentally, is not easy to be obtained in intra oral working conditions where curing might be less effective, which has been lately noticed (58). In addition, as an invasive method was applied for marginal evaluation, the adhesive marginal quality was measured on slices prepared by cross-sectioning. The three-dimensionality of all marginal and internal interfaces is, therefore, only partly represented by the obtained sections (31,47). It may be argued that a greater sample size, cutting the specimens in different areas would result in more discrimination in leakage analysis.

The study limitations include the fact that the teeth assignment was randomized with respect to the material to be tested, but not with respect to tooth morphology or location. These limitations should be considered when interpreting the results.

A major advantage of this setup is that the experimental testing conditions have permitted to benefit from standardized cavity dimension where high configuration factor (C-factor of 5) was almost identical between groups and all margins were confined to the enamel only.

Finally, the validity of leakage tests, as well as their correlation with the clinical performance adhesive restorative materials, have been questioned (59). It should be pointed, that indeed clinical outcome is not predicTable from marginal integrity alone (60). Although the arguments reported can be of scientific relevance, the practical and ethical need of preclinical *in vitro* investigations cannot be questioned (61). Among different approaches to predict clinical behavior such as microleakage assessment, thermocycling, mechanical loading, and subsequent marginal analysis may be the closest to the clinical situation (60,61).

To date there is no standardized procedure for assessing marginal integrity, however dye penetration is a well-established laboratory method for investigating marginal seal (44). Several tracer dyes have been suggested for microleakage studies, such as silver nitrate, fuchsin and methylene blue, but no significant difference in dye penetration among them have been reported (62). Even if some authors advocated that methylene blue may contribute to an overestimation of a leakage, this tracer dye has been described as suiTable for marking marginal gaps and was used in the present study (44).

Considering the experimental conditions of this *in vitro* study, it is possible to conclude that the application mode of the UA tested, rather than the investigated nanohybrid bulk-fill composite and the conventional nanohybrid composite, was the determining factor for marginal seal. This findings allowed to suggest the ER mode to increase the marginal seal of the UAs when full-body bulk-fill composite resins are used. Within the evaluated materials, the marginal quality of the nanohybrid bulk-fill composite placed in one increment up to 4 mm was comparable to that of the conventional nanohybrid composite placed in two increments, when same adhesive strategy was applied. Thus, high viscosity bulk‑fill composites may represent reliable alternatives to conventional composites for restoring high C-factor cavities. Such evidence should advisably be strengthened by further laboratory investigations using a larger sample size and a method for ageing. Ideally, long-term clinical trials are required to fully assess the suggested results.
